# Acute lymphoblastic leukemia presenting with mesenteric ischemia 

**Published:** 2019

**Authors:** Timothy Krill, Michelle Baliss, Jenine Zaibaq, Hamza M. Abdulla, Sreeram Parupudi

**Affiliations:** 1 *Department of Gastroenterology and Hepatology, The University of Texas Medical Branch, Texas, USA*; 2 *Department of Internal Medicine, The University of Texas Medical Branch, Texas, USA *

**Keywords:** Mesenteric ischemia, Leukemia, Arterial occlusion

## Abstract

Malignancy can induce a hypercoagulable state and lead to an increased risk of thromboembolic events. The pathogenesis of the prothrombotic state in cancer is complicated but is thought to involve several mechanisms. Thrombosis predominantly affects the venous circulation and infrequently the arteries. Arterial occlusion as an initial manifestation of acute leukemia is unusual. This is a case of a 44-year-old male admitted with complete thrombotic occlusion of the superior mesenteric artery and treated with emergent thrombectomy. Hematologic work-up was consistent with a diagnosis of T-cell acute lymphoblastic leukemia. To our knowledge, this is the first case of complete occlusion of the superior mesenteric artery presenting as the initial manifestation of T-cell acute lymphoblastic leukemia.

## Introduction

 Acute lymphoblastic leukemia (ALL) is a malignancy of B and T-cell lymphoid precursors that predominantly affects the pediatric population (1). The clinical presentation of ALL is often insidious. Patients typically present with symptoms related to bone marrow and organ infiltration (2). For example, there are increased rates of infectious complications from neutropenia and hemorrhagic events related to thrombocytopenia (3). However, despite severe thrombocytopenia, thromboembolism can still occur in the setting of ALL (4). The mechanism behind clot formation in acute leukemia is unclear. It is thought to be caused by increased production of prothrombotic factors, reduction of anticoagulant proteins, and damage to vascular endothelial cells. The latter can trigger cytokine production and platelet activation (5,6). Despite the increased risk of thromboembolism in ALL, treatment recommendations are controversial given the possibility of hemorrhage due to concurrent thrombocytopenia. 

Venous thromboembolism (VTE) in ALL is uncommon in the early stages of the disease, with an estimated incidence of 1.4%. The incidence increases to 10.6% following chemotherapy induction (7). However, arterial thromboses are uncommon as a presenting manifestation of acute leukemia (8). Here, we report a case of mesenteric ischemia due to complete SMA occlusion as the initial manifestation of T-ALL. 

## Case Report

**Figure 1:**
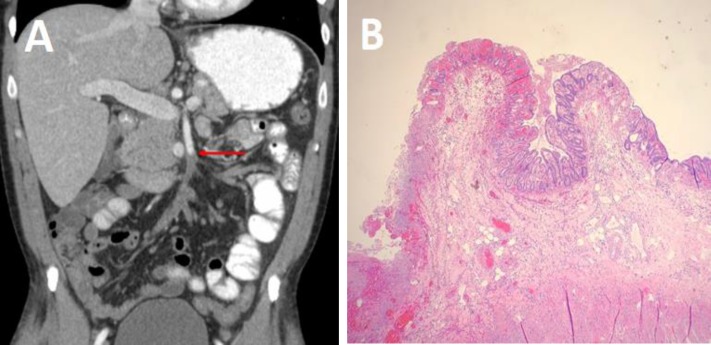
(A) Computed Tomography revealing a filling defect in the superior mesenteric artery (arrow) due to an acute thrombus. (B) Photomicrograph of resected small bowel tissue revealing vascular congestion, hemorrhage, and transmural necrosis

A 44-year-old male with no prior medical history presented with severe upper abdominal pain for one day along with nausea, vomiting, and diarrhea. Vital signs were unremarkable. The physical examination was notable for diffuse abdominal tenderness to palpation and pain out of proportion to exam. The respiratory, cardiovascular, and neurological exams were all normal. His initial laboratory studies were remarkable for a WBC of 2.02x10^3^/L with a blast count of 30%. The hemoglobin was 12.5g/dL, and the platelet count was 189,000/L. Lactic acid was within normal limits at 1.65 mmol/L. Computed tomography (CT) of the abdomen and pelvis with intravenous contrast revealed a thrombus in the SMA, resulting in complete occlusion (Figure 1). An emergent open SMA Thrombectomy was performed following the failure of catheter-directed thrombolysis. Diffuse ischemic changes were visualized in the jejunum necessitating a partial small bowel resection (Figure 1). An intraoperative transesophageal echocardiogram was performed to evaluate for a possible cardioembolic etiology; however, no intracardiac thrombus or valvular pathology was identified. 

**Figure 2:**
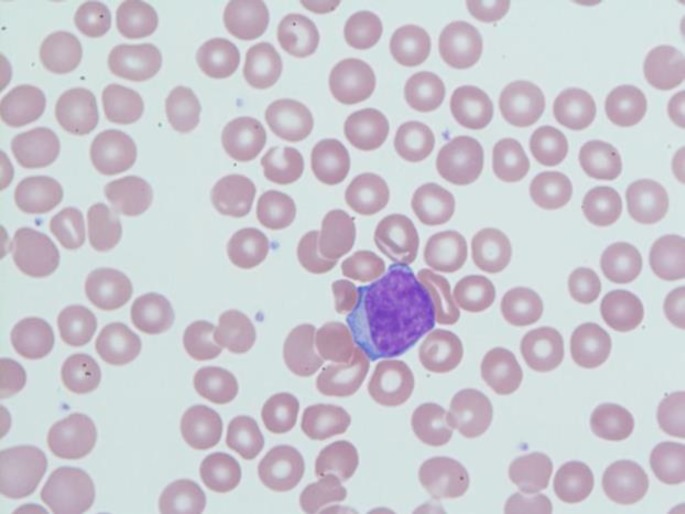
Peripheral blood smears revealing a lymphocyte with a high nuclear to cytoplasm ratio, loose chromatin, and an irregular nuclear shape consistent with a blast

Following thrombectomy, the patient was treated with a continuous infusion of heparin and ultimately transitioned to apixaban. Due to the initial complete blood count revealing blasts, further work-up was initiated. The peripheral blood smear revealed a large population of immature cells with fine chromatin and a high nuclear to cytoplasmic ratio (Figure 2). Flow cytometry demonstrated a large population of blasts constituting about 22% of cells. The cells were positive for CD5, CD7, and CD10. They were negative for surface CD3, CD4, CD8, CD17, and CD20. The immunophenotypic appearance was consistent with a diagnosis of T-cell ALL. He remained in stable condition postoperatively and was discharged with plans for outpatient bone marrow biopsy and follow-up with medical oncology to determine his plan of care. 

## Discussion

Thrombosis is a complication of acute leukemia with a significant impact on morbidity and mortality. Patients with underlying malignancy are at increased risk for thromboembolism through a multifactorial process. The inhibition of anticoagulant proteins, production of prothrombotic factors, alteration of the vascular endothelium to a thrombogenic state, and increased vascular adhesion of leukemic cells are all potential contributors (5,6,9).

VTE is a well-documented complication of acute leukemia. However, arterial occlusion is unusual and almost exclusively obsereved in acute promyelocytic leukemia (APL) (10). Additionally, the incidence of vascular thrombosis at the time of diagnosis in ALL is low at 1.4% (11). This contrasts with APL and non-M3 acute myeloid leukemia, in which 9.6% and 3.2% of patients are diagnosed with thromboembolism at the time of diagnosis, respectively (7,11).

Our patient’s case illustrates acute arterial occlusion in the SMA leading to mesenteric ischemia as the initial manifestation of T-cell ALL. SMA thromboembolism typically occurs in the setting of atherosclerosis or cardiac pathologies, such as arrhythmias or valvular vegetations (12). However, these were not present in this case. Thus, we believe the acute arterial thromboembolism in our patient occurred because of a hypercoagulable state precipitated by his underlying malignancy. Unfortunately, despite a successful thrombectomy and initiation of anticoagulation therapy, he suffered significant intestinal ischemia necessitating a partial small bowel resection. 

Most thromboembolic events in acute leukemia are related to chemotherapy. There are multiple reports of new thrombus formation or pre-existing thrombus extension during the induction phase of chemotherapy (6). L-asparaginase, which is a chemotherapeutic agent used to treat ALL, is believed to enhance thrombus formation by decreasing the levels of proteins C, S, and antithrombin III (13,14). It has also been shown in some studies to increase platelet aggregation (13). Certain agents also enhance endothelial activation as evidenced by increased plasma levels of P-selectin, von Willebrand factor antigen, and plasminogen activator inhibitor-1 (15).

Despite the high risk of thromboembolism in leukemic patients, there are no specific guidelines to direct management. In those determined to be at high risk for thrombosis, the prophylactic use of antithrombin, unfractionated heparin, or low molecular weight heparin (LMWH) may be effective in reducing the risk of VTE (16,17). The current approach for the treatment of VTE in acute leukemia centers on the use of LMWH (13,17). Anticoagulation management in ALL can be challenging, given the risk of bleeding due to thrombocytopenia. Decisions should be made on a case-by-case basis. In this patient, the benefits of anticoagulation outweighed the risks given the catastrophic small bowel necrosis that resulted from mesenteric ischemia. To our knowledge, this is the first reported case of complete SMA occlusion as a presenting manifestation of T-cell ALL. An unexplained arterial or venous thrombotic event in a previously healthy patient could represent the initial manifestation of an underlying malignancy. Induction chemotherapy can increase the risk of vascular occlusion by increasing platelet aggregation, enhancing endothelial activation, and disrupting the balance of pro- and anticoagulant proteins. The decision to initiate anticoagulation in patients with ALL complicated by thromboembolism needs to be made on a case-by-case basis in a multidisciplinary fashion.

## Conflict of interests

The authors declare that they have no conflict of interest.

## References

[1] Pui CH (2009). Acute lymphoblastic leukemia: introduction. Semin Hematol.

[2] Clarke RT, Van den Bruel A, Bankhead C, Mitchell CD, Phillips B, Thompson MJ (2016). Clinical presentation of childhood leukaemia: a systematic review and meta-analysis. Arch Dis Child.

[3] Villarreal-Martínez L, Jaime-Pérez JC, Rodríguez-Martínez M, González-Llano O, Gómez-Almaguer D (2012). Acute lymphoblastic leukemia of childhood presenting as aplastic anemia: report of two cases. Rev Bras Hematol Hemoter.

[4] Wu YY, Tang L, Wang MH (2017). Leukemia and Risk of Venous Thromboembolism: A Meta-analysis and Systematic Review of 144 Studies Comprising 162,126 Patients. Sci Rep.

[5] Caine GJ, Stonelake PS, Lip GY, Kehoe ST (2002). The hypercoagulable state of malignancy: pathogenesis and current debate. Neoplasia.

[6] Del Principe MI, Del Principe D, Venditti A (2017). Thrombosis in adult patients with acute leukemia. Curr Opin Oncol.

[7] De Stefano V, Sorà F, Rossi E, Chiusolo P, Laurenti L, Fianchi L (2005). The risk of thrombosis in patients with acute leukemia: occurrence of thrombosis at diagnosis and during treatment. J Thromb Haemost.

[8] Liu Y, Chao X, Gu W, Hua X, Xu N (2008). Acute thrombosis in superior mesenteric artery as first symptom in a AML patient. Int J Gen Med.

[9] Qureshi W, Ali Z, Amjad W, Alirhayim Z, Farooq H, Qadir S, Khalid F, Al-Mallah MH (2016). Venous Thromboembolism in Cancer: An Update of Treatment and Prevention in the Era of Newer Anticoagulants. Front Cardiovasc Med.

[10] D'Angelo P, Taormina C, Mosa C, Di Marco F, Valentino F, Trizzino A (2016). Severe Lower Limb Ischemia by Massive Arterial Thrombosis Revealing an Acute Myeloid Leukemia Needing for Leg Amputation: Clinical and Emotional Aspects Related to the Communication with the Patient and His Family. Pediatr Rep.

[11] Ku GH, White RH, Chew HK, Harvey DJ, Zhou H, Wun T (2009). Venous thromboembolism in patients with acute leukemia: incidence, risk factors, and effect on survival. Blood.

[12] Bjornsson S, Resch T, Acosta S (2013). Symptomatic mesenteric atherosclerotic disease-lessons learned from the diagnostic workup. J Gastrointest Surg.

[13] Goyal G, Bhatt VR (2015). L-asparaginase and venous thromboembolism in acute lymphocytic leukemia. Future Oncol.

[14] Sibai H, Seki JT, Wang TQ, Sakurai N, Atenafu EG, Yee KW (2016). Venous thromboembolism prevention during asparaginase-based therapy for acute lymphoblastic leukemia. Curr Oncol.

[15] Giordano P, Molinari AC, Del Vecchio GC, Saracco P, Russo G, Altomare M (2010). Prospective study of hemostatic alterations in children with acute lymphoblastic leukemia. Am J Hematol.

[16] Hunault-Berger M, Chevallier P, Delain M, Bulabois CE, Bologna S, Bernard M (2008). Changes in antithrombin and fibrinogen levels during induction chemotherapy with L-asparaginase in adult patients with acute lymphoblastic leukemia or lymphoblastic lymphoma. CAPELAL Study Haematologica.

[17] Grace RF, DeAngelo DJ, Stevenson KE, Neuberg D, Sallan SE, Mourad YRA (2018). The use of prophylactic anticoagulation during induction and consolidation chemotherapy in adults with acute lymphoblastic leukemia. J Thromb Thrombolysis.

